# Association between *UCP2* A55V polymorphism and risk of cardiovascular events in patients with multi-vessel coronary arterial disease

**DOI:** 10.1186/1471-2350-14-40

**Published:** 2013-03-27

**Authors:** Luciana Gioli-Pereira, Paulo CJL Santos, Luisa S Sugaya, Noely E Ferreira, José Eduardo Krieger, Alexandre C Pereira, Whady A Hueb

**Affiliations:** 1 Heart Institute (InCor), Sao Paulo University Medical School, Av. Dr. Enéas de Carvalho Aguiar, 44 Cerqueira César, São Paulo - SP, 05403-000, Brazil

**Keywords:** UCP2, A55V polymorphism, Coronary artery disease, Diabetes

## Abstract

**Background:**

UCP2 (uncoupling protein 2) plays an important role in cardiovascular diseases and recent studies have suggested that the A55V polymorphism can cause UCP2 dysfunction. The main aim was to investigate the association of A55V polymorphism with cardiovascular events in a group of 611 patients enrolled in the Medical, Angioplasty or Surgery Study II (MASS II), a randomized trial comparing treatments for patients with coronary artery disease and preserved left ventricular function.

**Methods:**

The participants of the MASS II were genotyped for the A55V polymorphism using allele-specific PCR assay. Survival curves were calculated with the Kaplan–Meier method and evaluated with the log-rank statistic. The relationship between baseline variables and the composite end-point of cardiac death, acute myocardial infarction (AMI), refractory angina requiring revascularization and cerebrovascular accident were assessed using a Cox proportional hazards survival model.

**Results:**

There were no significant differences for baseline variables according genotypes. After 2 years of follow-up, dysglycemic patients harboring the VV genotype had higher occurrence of AMI (p=0.026), Death+AMI (p=0.033), new revascularization intervention (p=0.009) and combined events (p=0.037) as compared with patients carrying other genotypes. This association was not evident in normoglycemic patients.

**Conclusions:**

These findings support the hypothesis that A55V polymorphism is associated with UCP2 functional alterations that increase the risk of cardiovascular events in patients with previous coronary artery disease and dysglycemia.

## Background

Cardiovascular diseases are the leading causes of death among adults [1]. Many risk factors such as age, gender, lipid abnormalities, hypertension, smoking, and genetic factors influence the occurrence and progress of the different atherosclerotic processes and diabetes. Indeed, 70% of patients with acute myocardial infarction (AMI) have either diabetes or impaired glucose tolerance [2].

Reactive oxygen species (ROS) and inflammatory cells, particularly monocytes and macrophages, have a major role in the pathogenesis of atherosclerosis [1]. ROS are also associated with the pathogenesis of atherosclerotic macrovascular disease in diabetic patients. At least four molecular mechanisms have been implicated in glucose-mediated vascular damage (advanced glycation end-products, AGE, Protein kinase C, polyol and hexosamine pathway). All of them seem to be related to overproduction of superoxide by the mitochondrial electron-transport chain, as a response to hyperglycemia [3].

In this context, UCP2 (uncoupling protein 2), a physiological down-regulator of ROS, might play an important role in cardiovascular diseases. Several studies have already described that UCP2 has an anti-atherogenic effect in the vascular wall [4], improves tolerance to cardiac ischemia [5,6] and protects cardiomyocytes from oxidative stress-induced cell death [7]. This role of UCP2 was demonstrated in transplanted mice (UCP2 -/-), which have presented larger atherosclerotic lesions, increased nitrotyrosine staining, suggesting enhanced oxidative stress, and a significant increase in macrophage accumulation in vascular lesions [8].

Nowadays, the exact functions of UCP proteins are not fully established and may be different according to assayed specific tissue. In β cells, UCP2 over expression leads to decreased insulin secretion. Thus, UCP2 disturbances can be related to the causes and complications of type 2 diabetes mellitus, both related to increased ROS production.

The A55V polymorphism has been associated with energy metabolism misbalance [9], higher risk of type 2 diabetes, lower HDL, high atherogenic index [10] and lower levels of leptin [11]. Besides, other studies have already associated VV genotype to lower degree of uncoupling, more efficient energy utilization, lower fat oxidation and increased ROS production suggesting that A55V cause UCP2 dysfunction [12,13].

In this scenario, the main aim of this study was to investigate whether the A55V polymorphism is associated to cardiovascular events in a group of 611 patients enrolled in the MASS II [14,15], a study that compared therapeutic strategies for individuals with multi-vessel coronary artery disease (CAD).

## Methods

### Patient selection

Studied patients were selected from the prospective, randomized and controlled Medicine, Angioplasty, or are medical treatment, angioplasty or stent (percutaneous coronary intervention, PCI), and myocardial revascularization (coronary artery bypass grafting, CABG) in patients with multi-vessel stable CAD with preserved left ventricular function [14,15].

Methodological details were described by Hueb et al. Briefly, patients with angiographically documented proximal multivessel coronary stenosis of more than 70% by visual assessment and documented ischemia were considered for inclusion. Ischemia was documented by either stress testing or the typical stable angina assessment of the Canadian Cardiovascular Society (CCS) (class II or III). Patients were enrolled and randomized if there was agreement on the part of the surgeon and interventionist that revascularization could be attained by either strategy. Exclusion criteria included unstable angina or acute MI requiring emergency revascularization, ventricular aneurysm requiring surgical repair, left ventricular ejection fraction of <40%, a history of PCI or CABG, and single-vessel disease. Patients were also excluded: if they had a history of congenital heart disease, valvular heart disease, or cardiomyopathy; if they were unable to understand or cooperate with the protocol requirements or to return for follow-up; or if they had left main coronary artery stenosis of 50% or more, or suspected or known pregnancy or another coexisting condition that was a contraindication to CABG or PCI. Patients were then randomized to continue with aggressive medical therapy (MT) alone or to undergo PCI or CABG concurrently with MT [14,15].

Two thousand seventy-six candidates who had indications for myocardial revascularization were evaluated from May 1995 to May 2000. Of these, 611 patients were eligible and met all inclusion criteria to be randomly assigned to 1 of the 3 therapeutic groups: medical treatment (n = 203), PCI (n = 205), and CABG (n = 203). The primary end-point of this study was to compare the frequencies of major cardiac events, such as acute MI, overall mortality or refractory angina requiring revascularization procedures and stroke. The main conclusion of the MASSII was relatively low rates of death with all 3 treatment regimens during the first and second follow-up years.

All subjects gave informed consent, and the Ethics Committee of the Heart Institute of the University of Sao Paulo - Brazil approved the study.

### Data collection

The beginning of treatment was considered to be the randomized date and patients were studied by the intention to treat principle. The follow-up time for all patients in this study was 5 years with clinical visits conducted in a 6-month interval. The collected demographic and laboratory data included age, gender, angiographic findings and traditional risk factors, such as history of previous coronary events, hypertension, diabetes, BMI (body mass index), severity of angina, smoking status, and cholesterol and triglycerides profile. Blood samples were obtained from each participant at randomization.

Symptoms of angina were graded according to their severity and were considered refractory only when patients had been treated with full anti-ischemic therapies. Myocardial infarction was defined as the presence of significant new Q waves in at least 2 ECG leads or symptoms compatible to MI associated with creatinine kinase-MB elevations. Cardiac mortality was defined when patient died of MI.

Glycemic status was defined using effective WHO classifications at that time of the randomization. Briefly, diabetic patients should present classic symptoms of hyperglycemia and an abnormal blood test [plasma glucose concentration ≥7 mmol/L (or 126 mg/dL) or ≥11.1 mmol/L (or 200 mg/dL) 2 hours after a 75 g glucose drink]. Impaired glucose tolerance were diagnosed in patients with fasting plasma glucose <7.0 mmol/L (or 126 mg/dL) and 2 hour post 75 g glucose drink of ≥ 7.8 mmol/L or 140 mg/dL and <11.1 mmol/L (or 200 mg/dL). Patients with diabetes and impaired glucose tolerance composed the dysglycemic group.

### A55V genotyping

Genomic DNA was extracted from leukocytes in samples of whole blood following standard techniques [16]. Genotyping was performed with an Array Tape technology (Douglas Scientific, Alexandria MN). A modified allele-specific PCR assay as described by Myakishev et al. was used [17]. Briefly, PCR were performed with 50 ng of genomic DNA in a total volume of 10 μlL containing 5 pmol of each primer, 100 μmol dNTPs, 0.3 U Taq DNA polymerase (Lab Trade, Brazil). An average genotyping success rate of more than 95% was obtained and, as quality control, an average genotyping accuracy of more than 98% was observed by re-genotyping of 32 samples.

### Statistical analysis

Categorical variables were presented as percentage while continuous variables were presented as mean ± standard deviation. General characteristics, biochemical and cardiovascular event frequencies were analyzed using Student’s *t* test for continuous variables and the Chi-square test or Fisher’s test for categorical variables. Survival curves were constructed with the Kaplan–Meier method and differences between the curves were evaluated with the log-rank statistic. We assessed the relationship between baseline variables and composite end-point events using a Cox proportional hazards survival model. Hazard ratios (relative risks) with 95% confidence intervals (CI) demonstrate the risk for combined events. Multiple testing correction was not performed. A value of p≤0.05 was considered significant for comparisons. Statistical analyses were performed with SPSS 13.0 for Windows. The Hardy-Weinberg equilibrium was conducted with Haploview 4.0.

## Results

The genotypic distribution for the A55V polymorphism was 20.7% of AA (n=116), 47.8% of AV (n=267) and 31.5% of VV (n= 176). Genotypic distributions were in accordance with the predicted by the Hardy-Weinberg equilibrium.

The Ala/Ala (AA) and the Ala/Val (AV) genotypes were combined and compared with the Val/Val (VV) genotype group. There was no significant difference in baseline values between these groups (Table 1).

**Table 1:** Baseline clinical characteristics

	**AA+AV (n= 383)**		**VV (n= 176)**		
		**Mean±SD**		**Mean±SD**	**p**
Frequency (%)	68.5		31.5		
Gender (man, %)	67.6		69.3		0.690
Age (y)		59.2±9.3		60.5±8.8	0.581
Hypertension (%)	56.7		62.5		0.193
Diabetes (%)	26.9		25.7		0.951
Glucose intolerance (%)	20.0		20.0		0.790
Normal Glucose Tolerance (%)	53.1		54.3		
Dysglicemic (%)	46.9		45.7		
High triglycerides (%)	55.8		58.6		0.537
LogTG*		2.2±0.2		2.2±0.2	0.308
Total Cholesterol (mg/dL)		222.0±48.3		224.8±47.2	0.522
LDL (mg/dL)		146.9±43.2		147.7±44.8	0.837
HDL (mg/dL)		37.4±10.5		37.4±10.6	0.961
Metabolic Sd (%)	51.2		47.3		0.462
Total Metabolic Sd		2.6±1.1		2.7±1.1	0.498
Obesity (%)	20.9		21.7		0.841
Double-vessel (%)	40.5		42.6		0.632
Multi-vessel (%)	59.5		57.4		
Medical therapy (%)	32.4		33.0		0.935
PCI (%)	33.9		32.4		
CABG (%)	33.7		34.7		
Angina CCS 1 (%)	6.1		6.8		0.475
Angina CCS 2 (%)	64.0		58.2		
Angina CCS 3 + 4 (%)	29.8		34.9		
Current smoker (%)	31.6		29.0		0.534
AMI (inicial exams) (%)	45.4		44.9		0.904

* Tryglicerides values were log transformed before comparison.

PCI: percutaneous coronary intervention.

CABG: coronary artery bypass surgery.

CCS: Canadian Cardiovascular Society.

AMI: acute myocardial infarction.

From 611 eligible patients, 559 individuals were genotyped and followed-up. There were 260 dysglycemic patients (148 diabetic and 112 glucose intolerants) and 299 patients with normal glucose tolerance as shown in Table 1. There were no differences between normoglycemic and dysglycemic patients regarding to cardiovascular events incidence during the first two follow-up years (Table 2).

**Table 2:** Cardiovascular event incidences in the entire cohort of patients, in the dysglycemic patients and in the normoglycemic patients

**Entire cohort of patients**					
	**AA+AV (n= 383)**		**VV (n= 176)**		
	**Count**	**%**	**Count**	**%**	**p**
**After 2 years**					
Death	21	5.5	12	6.8	0.548
AMI	22	5.7	16	9.1	0.138
Death + AMI	39	10.2	26	14.8	0.113
Revascularization Intervention	28	7.3	21	11.9	0.073
Events	84	21.9	49	27.8	0.098
**Dysglycemic patients**					
	**AA+AV (n= 180)**		**VV (n= 80)**		
	**Count**	**%**	**Count**	**%**	**p**
**After 2 years**					
Death	13	7.2	8	10.0	0.514
AMI	7	3.9	9	11.3	*0.023*
Death + AMI	18	10.0	16	20.0	*0.032*
Revascularization intervention	11	6.1	13	16.3	*0.006*
Events	36	20.0	26	32.5	*0.020*
**Normoglycemic patients**					
	**AA+AV (n= 203)**		**VV (n= 96)**		
	**Count**	**%**	**Count**	**%**	**p**
**After 2 years**					
Death	8	3.9	4	4.2	0.765
AMI	15	7.4	7	7.3	0.904
Death + AMI	21	10.3	10	10.4	0.883
Revascularization Intervention	17	8.4	8	8.3	0.969
Events	48	23.6	23	24.0	0.831

AMI: acute myocardial infarction.

Death CV: cardiovascular death.

PCI: percutaneous coronary intervention.

p-values are for log-rank statistics of KM curve comparison of 2 year follow-up period.

Stratifying our analysis by glucose homeostasis, significant differences were found in the subgroup of patients with dysglycemia after two years of follow-up. Patients carrying the VV genotype had a higher incidence of AMI (p=0.026), death+AMI (p=0.033), necessity of a new revascularization intervention (p=0.009) and combined events (p=0.037) (Table 2). Curiously, differences according to genotypic groups after 5 years of follow-up were not observed in dysglycemic, except for the variable AMI (p=0.025). For the normoglycemic patient group, no significant difference was found according to genotypes (Table 2).

**Figure 1:**
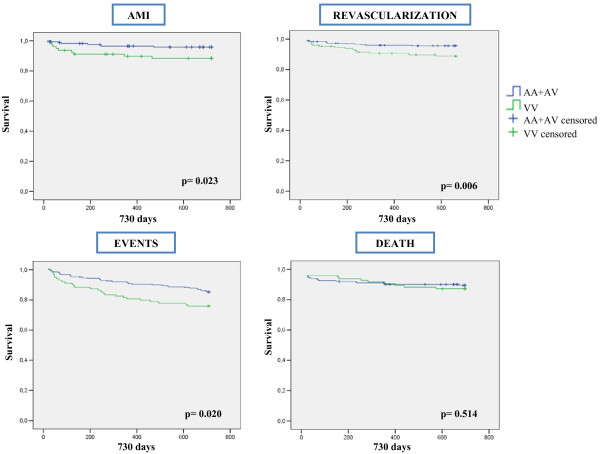
Survival curves of dysglycemic patients genotyped for the polymorphism A55V.

Similar results were found when Kaplan-Meier survival curves were constructed. There were significant differences for the incidence of AMI, new revascularization procedures and combined events as shown in Figure 1. Finally, modeling end-point events using a Cox proportional hazards model adjusted for age, sex, randomized treatment, and BMI, a significant interaction between dysglycemia and UCP2 genotype was observed for combined end-points (p = 0.03), incident myocardial infarction (p = 0.009), and new revascularization procedure (p = 023).

## Discussion

We investigated the relationship between the A55V polymorphism and the incidence of cardiovascular events in patients with CAD. Our main findings showed that A55V is associated to increased risk of cardiovascular events in patients with previous CAD and dysglycemia after two years of follow-up.

Other studies have already suggested that A55V polymorphism cause UCP2 dysfunction [12,13] supporting our findings. However, functional studies are necessary to confirm these affirmations and explain the association of VV genotype and cardiovascular events observed only in disglycemic patients. In this way, the CARDIA study reported the presence of diabetes in 5.8% of participants homozygous for the V allele which was statistically greater than the 3.3% and 3.7% in the AA and AV genotypes, respectively [13]. These results extend the findings from studies of Ala55Val or other UCP2 polymorphisms involved in the development of type 2 diabetes mellitus [18,19].

In our study, dysglycemic patients presented higher incidence of AMI, necessity of a new revascularization intervention and combined events, despite mortality being the same. Actually, it was described that functional alterations in UCP2 leads to a higher production of ROS that is already increased in dysglycemic patients and can worsen the macrovascular disease and the atherosclerotic process [3].

A study described that, in humans, the extent of uncoupling is partially regulated by the genetic polymorphism Ala55Val, in which the VV genotype uncouples at a lower rate than the AA genotype [20]. Other studies reported that the A allele was associated with the presence of higher oxidative stress markers and showed synergistic effect with other risk factors (obesity, hypertension and diabetes) [21,22].

There are some limitations in our study. First, we were not able to perform replication studies in a similar sample because there are very few studies similar to MASSII in the literature. Second, our sample size only provide 80% power to detect an association with death, AMI, revascularization intervention, events variables with an effect size of 1.7–2.4 or 1.8–3.6 for the A55V polymorphism in the total group and in the dysglycemic patients, respectively. We recognize that such effects are highly unlikely for common genetic variants and reaffirm the need for the development of increased samples of multivessel CAD patients with long-term follow-up period for the identification of more modest effects. Third, it is not possible to completely exclude the interaction between the use of concomitant drugs, other genetic markers, ethnicity and other covariates on our findings. We only analyzed the A55V polymorphism despite of others polymorphisms in the *UCP2* gene have already been identified and some of them are in linkage disequilibrium with A55V. For example, A866>G has already been associated to increased cardiovascular events risk, cardiometabolic disease and it represents a novel candidate gene for beta-blocker response, particularly among patients with diabetes [23].

A recent meta-analysis has showed that the *UCP2* -866G/A polymorphism is unlikely associated with increased type 2 diabetes risk in the Asian and European descent. In contrast, further results indicate that the *UCP2* Ala55Val and *UCP3* -55C/T polymorphisms may indeed be risk factors for susceptibility to type 2 diabetes in individuals of Asian descent, but not in individuals of European descent [24].

After 2 years of follow-up, dysglycemic patients carrying the VV genotype had higher incidence of AMI, death+AMI, new revascularization intervention and combined events compared to patients carrying other genotypes. After 5 years of follow-up, we were not able in detect these differences according to genotypic groups, except for AMI incidence. We speculate that the predictive effect of *UCP2* genotype in dysglycemic patients could be related to the time of illness. On the other hand, there is no experimental evidence suggesting that the duration of diabetes could play a role in this result. Further studies are necessary to establish the role of VV genotype as an independent genetic marker of cardiovascular events in this specific subgroup.

## Conclusion

Taken together, our findings corroborate the hypothesis that A55V polymorphism is associated with UCP2 functional alterations that may increase the risk of cardiovascular events in patients with previous CAD and dysglycemia.

## Abbreviations

UCP: Uncoupling protein 2; CAD: Coronary artery disease; MASSII: Medical angioplasty or surgery study II; AMI: Acute myocardial infarction; ROS: Reactive oxygen species; PCI: Percutaneous coronary intervention; CABG: Coronary artery bypass grafting; CCS: Canadian cardiovascular society; MT: Medical therapy; MI: Myocardial infarction; BMI: Body mass index; WHO: World health organization

## Competing interests

The authors declare that they have no competing interests.

## Authors’ contributions

LGP and LSS carried out part of the molecular genetic studies, performed the statistical analysis and drafted the manuscript. LGP and PCJLS performed the revision and statistical analysis of the manuscript. NEF carried out part of the molecular genetic studies. WAH and JEK conceived the study, and participated in its design. ACP conceived the study, participated in its design and coordination and helped to draft the manuscript. All authors read and approved the final manuscript.

## Pre-publication history

The pre-publication history for this paper can be accessed here:

http://www.biomedcentral.com/1471-2350/14/40/prepub
